# The association between food addiction, eating attitudes, self-esteem, and emotional appetite: a cross-sectional study

**DOI:** 10.3389/fpsyg.2025.1703745

**Published:** 2025-11-14

**Authors:** Nevin Sanlier, Omur Alyakut

**Affiliations:** 1 Department of Nutrition and Dietetics, School of Health Sciences, Ankara Medipol University, Ankara, Türkiye; 2 Kartepe Vocational School of Tourism, Kocaeli University, Kartepe, Türkiye

**Keywords:** young people, food addiction, self-esteem, emotional appetite, eating attitude

## Abstract

**Background:**

Eating behavior is a multidimensional construct shaped by physical, emotional, and psychological factors. This study investigated the relationships and effects of self-esteem, emotional appetite, and food addiction on eating attitudes among young adults, while also assessing the role of demographic variables in relation to eating attitudes (EAT-26 scores).

**Methods:**

Data were collected from 766 participants (171 women, 595 men) using the Eating Attitudes Test-26 (EAT-26), Rosenberg Self-Esteem Scale (RSB), Emotional Appetite Questionnaire (EMAQ), and Yale Food Addiction Scale (YFAS).

**Results:**

Among the participants, 71.7% had a healthy weight, 13.0% were underweight, and 15.2% were overweight or obese. Significant associations were observed between the EAT-26 eating preoccupation subscale and educational level, smoking, skipping snacks, eating out (*p* < 0.05), and body mass index (BMI) (*p* < 0.01). The restriction subscale was positively correlated with BMI and perceived adequate nutrition (*p* < 0.01), whereas the social pressure subscale was negatively correlated with educational status, BMI (*p* < 0.01), and skipping snacks (*p* < 0.05). The EAT-26 total score was significantly associated with self-esteem (RSB), negative emotional appetite (EMAQ negative), and food addiction (YFAS) (*p* < 0.01). Further analyses identified gender (*B* = −2.00; *p* < 0.05), negative emotional appetite (*B* = 0.03; *p* < 0.05), and food addiction (*B* = 0.496; *p* < 0.01) as significant predictors of EAT-26.

**Conclusion:**

The results indicate that eating behavior is most strongly influenced by food addiction, negative emotional appetite, and low self-esteem, underscoring the decisive role of psychological factors.

## 1 Introduction

Food addiction, eating attitudes, self-esteem, and emotional appetite are interrelated psychological factors that significantly influence eating behaviors, particularly among young adults. Low self-esteem, negative eating attitudes, and heightened emotional appetite have been associated with an increased risk of food addiction and eating disorders. The reported prevalence in the literature ranges from 13.2 to 18.2% (Borisenkov et al., 2020; Bozkurt et al., 2024), alongside a documented rise in disordered eating attitudes and behaviors (Paterna et al., 2021).

Depression and stress increase the tendency toward uncontrolled eating, which arises as a way of coping with negative emotions. This tendency is significantly associated with symptoms of eating addiction (EA) (Fernández et al., 2022). Studies have shown that eating addiction has a positive correlation with mental health symptoms such as eating disorders, depression, and anxiety (Burrows et al., 2018; Cinelli et al., 2020). The emergence of the healthy and clean eating movement has contributed, in some cases, to an oppression with the consumption of high-quality, health-oriented foods. When this oppression escalates, it can result in pathological orthorexic behaviors and restrictive dietary practices, a condition known as orthorexia nervosa (ON). Despite its prevalence, the relationship between eating behavior, emotional appetite, and food addiction is complex and multifaceted. It has been reported that individuals with low self-esteem are more likely to exhibit maladaptive eating behaviors as a coping mechanism for emotional distress (Sanlier et al., 2017).

Emotional states significantly influence eating behavior, with factors such as self-esteem, impulse control, and emotional coping mechanisms playing a particularly decisive role among young adults. Emotional appetite refers to the tendency to eat in response to emotional states rather than physiological hunger. Negative emotional states such as stress and depression are associated with uncontrolled eating and eating as a coping mechanism (Fernández et al., 2022; Rossi, 2025). Eating disorders are associated with higher body mass index, emotional eating, and uncontrolled eating behaviors (Sanlier et al., 2017; Sanlier et al., 2017). Another study reported that food addiction elevates the risk of eating disorders, identifying body mass index (BMI) as the most influential factor. The findings further indicated that women are more likely than men to experience both anorexia nervosa and food addiction (Rose et al., 2018). Emotional eating and delicious eating motivations are reported to be more common among individuals who had food addictions (FA) (Bozkurt et al., 2024). Young people with a stronger emotional appetite often engage in uncontrolled eating behaviors, potentially worsening food addiction (Rossi, 2025). Within the brain reward system, dopamine, which plays a role in the development of addiction and is the most important neurotransmitter, comes to the fore. D2 receptors, which are elements of the dopaminergic system, are affected by sugar consumption. Sugar addiction creates the sense of pleasure through the brain’s reward system. Long-term sugar consumption seems to reduce the level of D2 receptors. Acute sugar consumption results in intense opioid and dopamine release. Furthermore, acetylcholine release is delayed, resulting in increased food intake. Chronic sugar consumption results in dopamine deficiency and dysfunction in the prefrontal cortex, the reward center. When sugar consumption is absent, withdrawal symptoms develop, leading to compulsive eating disorder (DiNicolantonio et al., 2018). There appears to be a significant overlap between added sugar consumption and drug-like effects such as overeating, cravings, tolerance, withdrawal, cross-sensitization, cross-tolerance, cross-dependence, reward, and opioid effects, including sugar addiction and dependence on the natural endogenous opioids released by sugar intake (Serin and Şanlıer, 2018). Processed foods are made palatable by adding large amounts of fat, sugar, or salt and are widely consumed. Therefore, these foods promote overeating, which is the leading cause behind obesity and associated metabolic diseases (Bozkurt et al., 2024; Bjorlie et al., 2022; Gearhardt and DiFeliceantonio, 2023).

Among adolescents with morbid obesity, negative moods combined with difficulties in impulse control, emotional eating, and food addiction are linked to a lower quality of life. These emotional regulation strategies have been shown to be crucial in managing eating behaviors and preventing food addiction (Rose et al., 2018). They might perceive themselves as overweight. This issue highlights the relationship between body image and eating behaviors (Lipsky et al., 2022).

One study found positive correlations between emotional eating and food addiction scores among men and women, and psychological distress scores were positively correlated with body mass index (BMI). Among educated young adults, the use of food consumption to regulate negative moods has been reported to put individuals at risk for overweight and obesity (Bourdier et al., 2018). In addition, it has been reported that eating disorders such as cognitive restraint, uncontrolled eating, and emotional eating also contribute to an increased risk of food addiction among students (Alim et al., 2021). In addition, the promotion of an idealized body image within the media influences societal perceptions, equating thinness with beauty. Inconsistencies between one’s desired and actual body lead individuals to feel anxious about their bodies and develop body dissatisfaction over time. As distorted body perceptions cause psychological distress, they are effective in the development of disordered eating behaviors (Paterna et al., 2021; Sahin and Sanlier, 2025). These findings highlight the complex interactions between emotional states, eating behaviors, and food addiction among young adults. Ultimately, these dynamics highlight the importance of examining psychosocial factors that contribute to the likelihood of developing eating disorders.

This study aims to investigate the relationships between self-esteem (RSB), emotional eating (EMAQ), food addiction (YFAS), and eating attitudes (EAT-26) among young adults aged 20 to 30 years old. It also examines how demographic factors are related to EAT-26 scores and the extent to which they may influence these attitudes.

## 2 Materials and methods

### 2.1 Procedure and participants

For this cross-sectional study, a total of 766 adult volunteers, including 171 (22.3%) women and 595 (77.7%) men aged 20–30 living in Ankara (the capital of Turkey), were reached. A total of 915 people were sent the study link. However, only 766 of them completed the survey completely and accurately, resulting in an 83.7% response rate. The study was carried out between November 2024 and March 2025. The questionnaire package prepared by the researchers was sent to the participants via Google Forms links shared on WhatsApp, Instagram, and Facebook, which are connected to the researchers’ systems. Data were collected using the convenience sampling technique. Before implementing the questionnaire package, a pilot study was conducted among 48 participants, and unexplained points were examined and minor modifications and adjustments were made to questions that were unclear. Participants under the age of 20 or over the age of 30, those who were pregnant or breastfeeding, and those with mental illness were not included in the research. To ensure the survey’s comprehensibility and accuracy, participants with mental illness, pregnant women, and breastfeeding women were excluded from the study.

This study aimed to investigate the relationships and predictive effects of self-esteem, emotional appetite, and food addiction on eating attitudes among young adults. In addition, it examined the associations and potential influences of demographic characteristics on EAT-26 scores.

The hypotheses formulated in line with the study objective are as follows:

Demographic characteristics are significantly associated with eating attitudes.

Self-esteem is significantly associated with eating attitudes.

Emotional appetite is significantly associated with eating attitudes.

Food addiction is significantly associated with eating attitudes.

**Figure 1:**
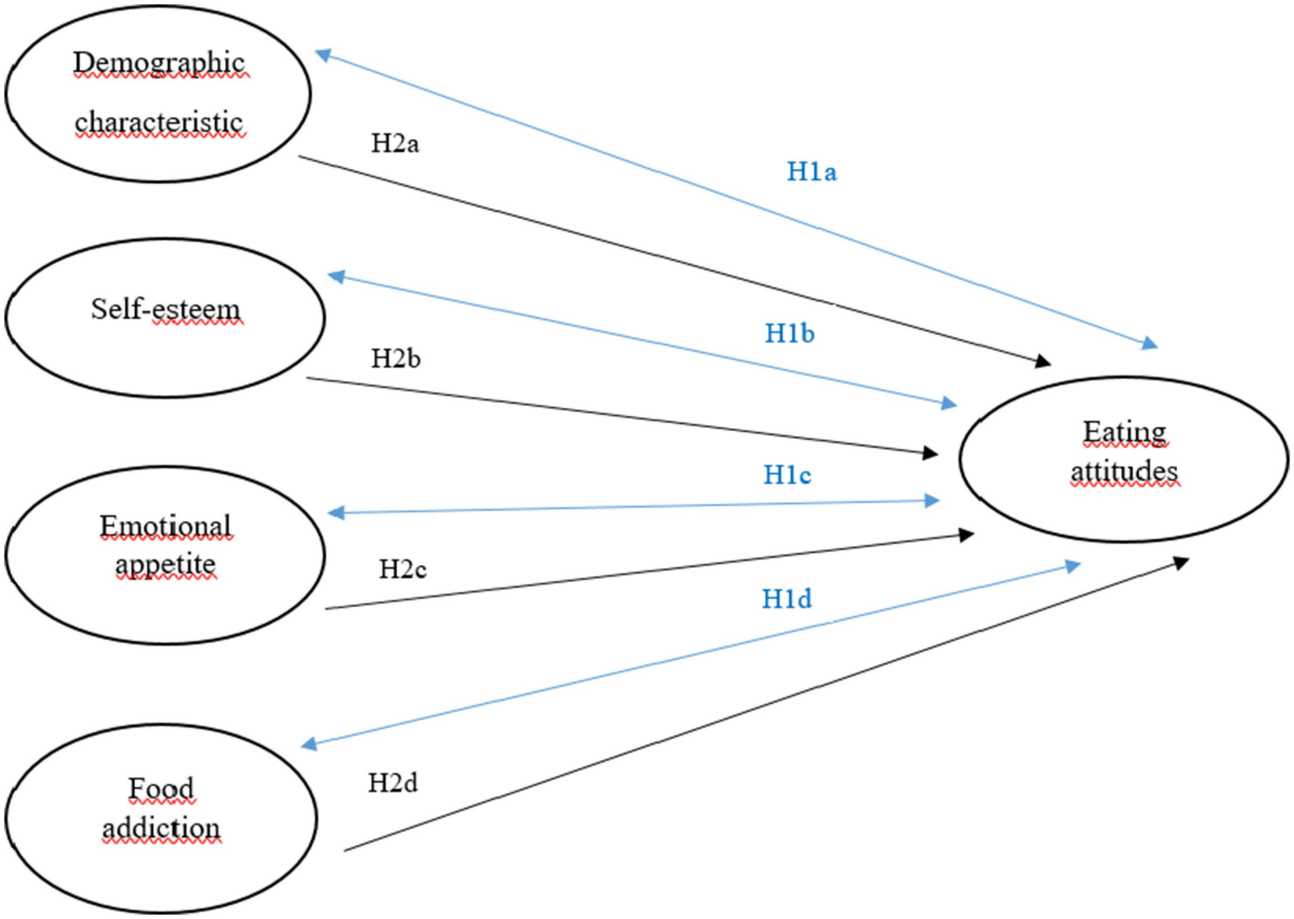
Hypothesized research model.

The research model containing all the hypotheses proposed in the study is presented in Figure 1.

### 2.2 Demographic information

Questions about sociodemographic characteristics such as gender (male/female), age (years), and length of education (years), as well as current smoking status (yes/no) and the presence of any health problems (yes/no) were provided. Additionally, participants reported their height (cm) and body weight (kg) based on self-reported information.

### 2.3 Instrumentation

The questionnaire, after revision, was arranged into four distinct sections:

1. Rosenberg Self-Esteem Scale (RSES) (10 statements) (Gearhardt and DiFeliceantonio, 2023)
2. Yale Food Addiction Scale (YFAS) (25 statements) (Lipsky et al., 2022)
3. Eating Attitudes Test (EAT-26) (26 statements) (Bourdier et al., 2018)
4. Emotional Appetite Questionnaire (EMAQ) (22 statements) (Alim et al., 2021).

#### 2.3.1 Rosenberg self-esteem scale (RBS)

The Rosenberg Self-Esteem Scale, originally developed by Rosenberg (1965), was employed in this study to assess self-esteem. This self-report instrument comprises 63 multiple-choice items divided into 12 subscales, each of which may also be applied independently in research. The Self-Esteem Subscale of the RBS Scale is a 4-point Likert-type scale that comprises the first 10 items of the scale. These 10 items were used in this study. Since responses are scored in single, double, or triple groups, the total score range of the subscale is 0–6. According to Rosenberg (1965), individuals scoring between 0 and 1 points are considered to have high self-esteem, those with scores ranging from 2 to 4 are regarded as having moderate self-esteem, and those scoring 5 to 6 points are identified as having low self-esteem.

#### 2.3.2 Yale food addiction scale

The Yale Food Addiction Scale (YFAS) was developed by Gearhardt et al. (2009). This scale consists of 27 items that assess participants’ food addiction status by inquiring about seven criteria related to their eating habits over the past 12 months. The first 18 questions of the scale are five-point Likert-type questions. However, responses to questions 19–24 are either “yes.” or “no.” Information on how many times an individual has attempted to reduce or stop eating certain foods in the past year is obtained from question 25, while information on foods that individuals find difficult to control is obtained from questions 26 and 27. Questions 17, 18, and 23, which are prerequisites for other questions, are not included in the scoring. Each question is scored as 0 or 1 based on the response given to the items. Subsequently, the total score for the questions under each dependency criterion (tolerance, withdrawal, clinical significance, etc.) is calculated. The degree of addiction is proportional to the number of symptoms. Questions 15 and 16 are clinically significant in identifying food addiction, and those who scored at least 1 point on these questions and had 3 or more symptoms were defined as “food addicts” (Gearhardt et al., 2009). In addition to providing a continuous symptom count, the YFAS also yields a dichotomous diagnostic classification. The Cronbach’s alpha coefficient of the scale used in this study was found to be 0.811.

#### 2.3.3 Eating attitudes test-26 (EAT-26)

The Eating Attitudes Test (EAT-26) assesses the risk of eating disorders by evaluating eating-related attitudes, emotions, and behaviors. Scores range from 0 to 78, and a cutoff score of ≥20 is used. Higher scores reflect an increased risk of eating disorders. Total scores of 20 or higher are considered to be in the clinical range. Individuals scoring 20 or higher are classified as “At Risk for Eating Disorders,” while those scoring below 20 are classified as “Not At Risk for Eating Disorders.” Responses are scored as follows: always = 3, usually = 2, often = 1, other options = 0. Only item 26 is reverse-scored: never = 3; rarely = 2; sometimes = 1; other options = 0 (Garner and Garfinkel, 1979).

#### 2.3.4 Emotional eating scale (EMAQ)

The scale consists of 22 items and does not include a cut-off score. A Likert-type scale was used, designed according to a 9-point scoring system (Nolan et al., 2010). In the EMAQ, emotional eating is evaluated through 14 items addressing positive and negative emotions and 8 items focusing on both positive and negative situations. If an individual consumes significantly less food than normal in certain emotions and situations, they mark numbers 1–4; if there is no change in food consumption, they mark number 5; if they consume significantly more food than normal, they mark numbers 6–9. The EMAQ negative score is calculated by summing responses to negative emotions and situations, while the positive score is derived from responses to positive emotions and situations.

### 2.4 Anthropometric measurements

Information regarding participants’ height and body weight was obtained based on their self-reports. The body mass index (BMI) was calculated by dividing body weight by the square of height and evaluated using the World Health Organisation’s BMI classification. Body weight (kg), and the obtained data were divided into three groups: underweight (BMI < 18.5 kg/m^2^), healthy weight (18.5 ≤ BMI ≤ 24.9 kg/m^2^) and overweight (BMI ≥ 25.0 kg/m^2^) (World Health Organization, 2010; World Health Organization, 2000).

All participants were fully informed about the purpose of the study prior to completing the questionnaire through the system, and informed consent was obtained in line with the principles of the Declaration of Helsinki (World Medical Association). It took approximately 20 min to complete the survey.

### 2.5 Data analysis

Artificial Neural Network method, a subtype of artificial intelligence and machine learning, was used in the research. Although artificial neural network analysis is a topology research method developed for operations research, unlike other operations analysis, it is also used in applications of decision selection problems in different fields. The method is based on producing nonlinear predictions and provides more compatible results in complex data and models with linearization deviations. An analysis used in the study is, it is an Artificial Neural Network method, which is a subtype of artificial intelligence and machine learning. Although artificial neural network analysis is a topology research method developed for operations research, unlike other operations analysis, it is also used in applications of decision selection problems in different fields. The method is based on producing non-linear predictions and provides more compatible results in complex data and models with linearization deviations. With the artificial neural networks method, clustering and desired classification levels are higher, and multi-layered and efficient results can be obtained in multivariate decision and prediction analyses. In this context, a multilayer perceptron model was applied for the Artificial Neural Network analysis used in the study. All analyses were carried out in the SPSS 25.0 program with a 95% Confidence Interval and a significant level of 0.05.

## 3 Results

A total of 766 volunteers, including 171 females and 595 males, participated in this descriptive cross-sectional study. The majority of participants were male (77.7%). The highest rates participation was from the health sciences (58.2%) departments, followed by tourism (19.1%) department and education (18.5%) departments. In reaction to the BMI, 13.0% of the participants were underweight, 71.8% had a healthy weight, and 15.2% of the participants were overweight/obese. In addition, 14.5% of the participants were smoking and 14.5% were drinking alcohol. Within the whole sample, 43.2% of the participants thought that they have an adequate, balanced, healthy diet, and 97.0% of them stated that they eat out, and 57.4% of them eat at school or workplace restaurants, the majority skip breakfast or lunch, 71.5% eat out of home 1–6 times a week, and 58.1% eat at school-work restaurants (Table 1).

**Table 1:** Baseline characteristics of participants (*n* = 766).

**Characteristics**		***n***	**%**
Gender	Male	595	77.7
	Female	171	22.3
Faculty	Health sciences	446	58.2
	Education	142	18.5
	Tourism	146	19.1
	Engineering and management	32	4.2
BMI (kg/m^2^)	Under weight	100	13.0
	Healthy weight	550	71.8
	Overweight	116	15.2
Smoking	No	655	85.5
	Yes	111	14.5
Alcohol	No	655	85.5
	Yes	111	14.5
Balanced, healthy diet	No	435	56.8
	Yes	331	43.2
Skipping main meal	No	215	28.1
	Sometimes	335	43.7
	Yes	216	28.2
Type of skipped meal	Breakfast	245	44.5
	Lunch	283	51.4
	Dinner	23	4.1
Eating out	No	23	3.0
	Yes	743	97.0
Type of meal eaten out	Breakfast	46	6.2
	Lunch	526	70.8
	Dinner	171	23.0
Eating out (frequency)	1–3 times in a month	46	6.2
	1–6 times a week	531	71.5
	Everyday	166	22.3
Eating out (place)	School-job restaurant	432	58.1
	Restaurant	71	9.6
	Kebap house	53	7.1
	Fast-food	187	25.2

The internal consistency of the scales employed in the study was assessed using Cronbach’s alpha coefficient. High reliability levels were observed in the EMAQ negative (*α* = 0.862), EMAQ positive (*α* = 0.841), YFAS (*α* = 0.811), and EAT-26 Eating Obsession (*α* = 0.819) subscales. The reliability level for RSB (*α* = 0.729) and EAT-26 Total (*α* = 0.773) was found to be acceptable. The reliability level for the EAT-26 Restriction and Social Pressure subscales was moderate, with *α* = 0.641. Participants’ RSB scores ranged from 10.0 to 55.0, with a total score average of 23.2 ± 4.63. The EMAQ negative average was 59.8 ± 21.05, and the EMAQ positive average was 43.5 ± 12.73. The total YFAS average is 5.4 ± 4.40. EAT-26 total scores range from 0.0 to 51.0, with an average of 21.49 ± 8.28 (Table 2).

**Table 2:** Descriptive statistics for scale scores with Cronbach Alpha internal consistency level.

Scales	Cronbach alpha	Minimum	Maximum	Mean	SD
RSB total	0.729	10.0	40.0	23.27	4.63
EMAQ negative	0.862	12.0	150.0	59.87	21.05
EMAQ positive	0.841	3.0	88.0	43.55	12.73
YFAS total	0.811	0.0	26.0	5.49	4.40
EAT-26 dieting	0.819	0.0	28.0	10.03	5.72
EAT-26 bulimia and food preoccupation	0.641	0.0	17.0	6.62	2.98
EAT-26 oral control	0.641	0.0	12.0	4.84	2.68
EAT-26 total	0.773	0.0	51.0	21.49	8.28

RSB: Rosenberg Self Esteem Scale, EMAQ: The Emotional Appetite Questionnaire, YFAS: The Yale Food Addiction Scale, EAT-26: Eating Attitudes Test Short Form.

The preoccupation with eating sub-scale of EAT-26 was significantly correlated with the participants’ faculty (*r* = 0.080; *p* < 0.05), BMI (*r* = 0.246; *p* < 0.01), smoking (*r* = 0.084; *p* < 0.05), skipped snack (*r* = 0.092; *p* < 0.05), and eating out (place) (*r* = 0.086; *p* < 0.05). The restriction subscale of EAT-26 was significantly correlated with BMI (*r* = 0.100; *p* < 0.01) and adequate nutrition (*r* = 0.108; *p* < 0.01). The social pressure sub-scale of EAT-26 was significantly correlated with the participants’ faculty (*r* = −0.094; *p* < 0.01), BMI (*r* = −0.345; *p* < 0.01), and skipping snacks (*r* = −0.080; *p* < 0.01). EAT-26 total was significantly correlated with gender (*r* = 0.083; *p* < 0.05) and BMI (*r* = 0.083; *p* < 0.05) (Table 3).

**Table 3:** Correlation between EAT-26 and demographic characteristics.

Demographic characteristics	Dieting	Bulimia and food preoccupation	Oral control	EAT-26 SF total
Gender	0.036	0.048	0.044	**0.083** ^*^
Faculty	**0.080** ^*^	−0.064	**−0.094** ^**^	−0.002
BMI	**0.246** ^**^	**0.100** ^**^	**−0.345** ^**^	**0.083** ^*^
Smoking	**0.084** ^*^	0.024	0.028	0.063
Alcohol	0.026	0.015	0.016	0.024
Adequate nutrition	−0.018	**0.108** ^**^	−0.038	0.005
Skipping main meal	−0.007	−0.062	0.028	−0.013
Skipped main meal	0.066	0.067	−0.052	0.047
Skipping snack	0.016	−0.038	**−0.080** ^*^	−0.025
Skipped snack	**0.092** ^*^	0.013	−0.004	0.056
Eating outside	−0.019	−0.033	0.011	−0.020
Outside meal	0.013	−0.007	−0.047	−0.016
Eating outside frequency	−0.017	−0.065	0.043	−0.015
Eating outside place	**0.086** ^*^	−0.019	0.012	0.046

*p < 0.05, **p < 0.01, BMI: Body Mass Index, EAT-26: Eating Attitudes Test Short Form.

There were statistically significant correlations between the preoccupation with eating subscale and RSB total (*r* = −0.149; *p* < 0.01), EMAQ negative (*r* = 0.222; *p* < 0.01), and YFAS total (*r* = 0.466; *p* < 0.01). The restriction subscale was significantly correlated with EMAQ positive (*r* = −0.141; *p* < 0.01). The social pressure sub-scale was significantly correlated with EMAQ negative (*r* = −0.088; *p* < 0.01) and EMAQ positive (*r* = 0.149; *p* < 0.01). The EAT-26 total score was significantly correlated with RSB total (*r* = −0.103; *p* < 0.01), EMAQ negative (*r* = 0.133; *p* < 0.01), and YFAS total (*r* = 0.323; *p* < 0.01) (Table 4).

**Table 4:** Correlation between EAT 26 and RSB, EMAQ, and YFAS scales.

Scales	Dieting	Bulimia and food preoccupation	Oral control	EAT-26 total
RSB total	**−0.149** ^**^	0.032	−0.020	**−0.103** ^**^
EMAQ negative	**0.222** ^**^	0.036	**−0.088** ^*^	**0.133** ^**^
EMAQ positive	−0.069	**−0.141** ^**^	**0.149** ^**^	−0.024
YFAS total	**0.466** ^**^	−0.070	0.054	**0.323** ^**^

*p < 0.05, **p < 0.01. RSB: Rosenberg Self Esteem Scale, EMAQ: The Emotional Appetite Questionnaire, YFAS: The Yale Food Addiction Scale, EAT-26: Eating Attitudes Test Short Form.

Generalized linear model at multivariable level showed that the effects of gender (*B* = −2.00), EMAQ negative (B = 0.03; *p* < 0.05), and YFAS total (B = 0.496; *p* < 0.01) on EAT-26 were statistically significant (Table 5).

**Table 5:** Generalized linear model (scale logit) for effects of correlated factors on EAT-26 SF total score.

Parameter	*B*	Std. error	95% Wald confidence ınterval		Hypothesis test		
			Lower	Upper	Wald chi-square	df	*p-*value
(Intercept)	19.85	2.68	14.61	25.10	55.00	1	0.000
[Gender = Female]	−2.00	0.71	−3.39	−0.62	8.04	1	0.005
[Gender = Male]	0^a^	.	.	.	.	.	.
[BMI = Underweight]	−2.18	2.14	−6.36	2.01	1.04	1	0.308
[BMI = Healthy weight]	−0.52	2.00	−4.44	3.40	0.07	1	0.794
[BMI = Overweight]	−0.53	2.11	−4.67	3.61	0.06	1	0.802
[BMI = Obese]	0^a^	.	.	.	.	.	.
RSB Total	−0.08	0.06	−0.20	0.05	1.50	1	0.221
EMAQ Negative	0.03	0.01	0.01	0.06	5.22	1	0.022
YFAS Total	0.50	0.07	0.36	0.63	53.24	1	0.000
(Scale)	61.38^b^	3.14	55.52	67.86			

Dependent Variable: EAT26_Total. Model: (Intercept), Gender, BMI, RSB_Total, EMAQ_Negative, YFAS_Total. ^a^Set to zero because this parameter is redundant, ^b^Maximum likelihood estimate. RSB: Rosenberg Self Esteem Scale, EMAQ: The Emotional Appetite Questionnaire, YFAS: The Yale Food Addiction Scale.

**Figure 2:**
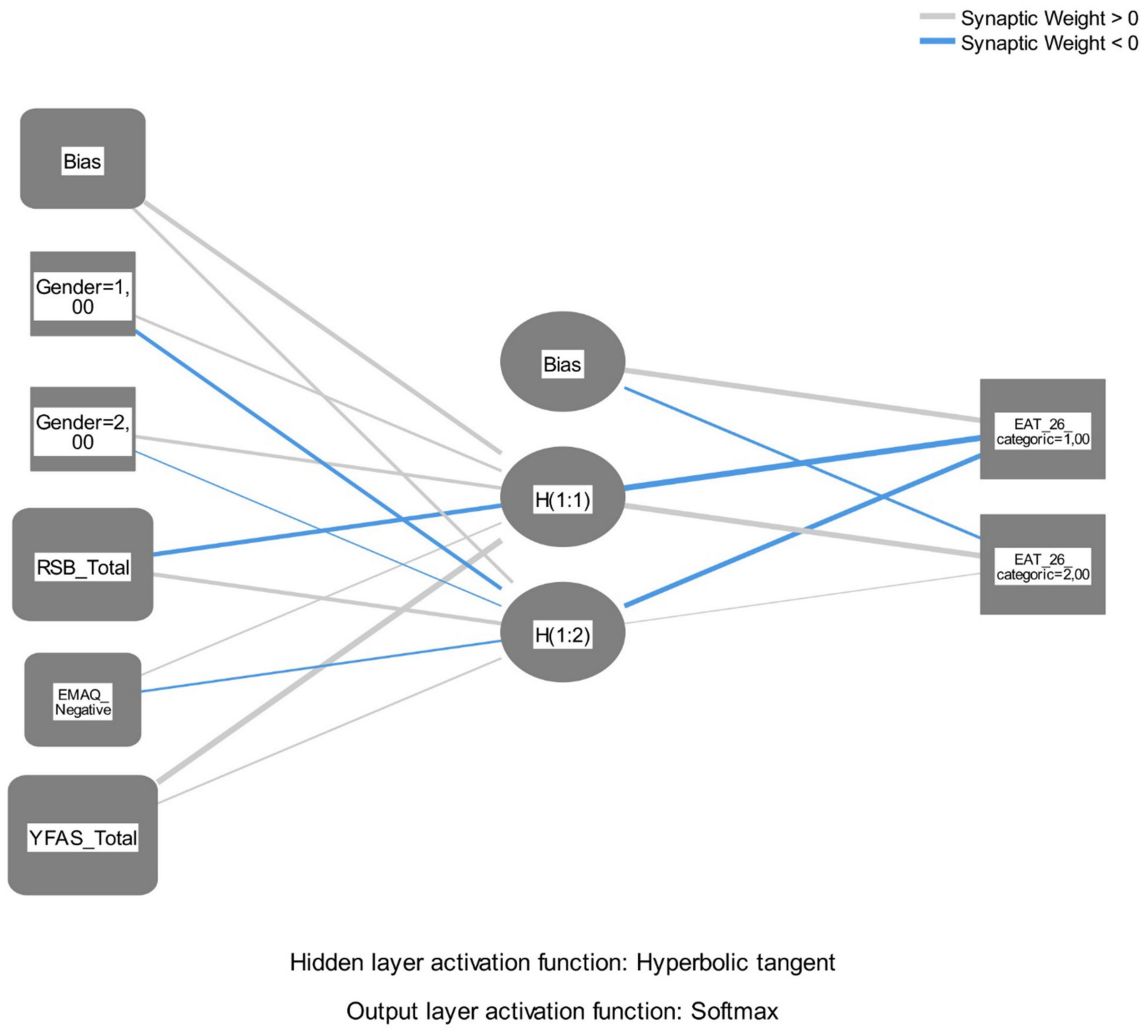
Artificial neural networks multilayer perceptron path results.

Both synaptic weights over and under zero results showed artificial neural network results consistency (Figure 2).

Hidden layer parameter values showed that YFAS had the highest value (0.950) followed by RSB (−0.538; absolute value) and EMAQ negative (0.302) in H(1:1) layer. In H(1:2) layer, RSB (0.536) had the highest value, followed by EMAQ negative (0.371; absolute value) and YFAS (0.340) (Table 6). According to the model summary output, the artificial neural network correctly classified 62.9% of the training data and 64.9% of the testing data, corresponding to incorrect prediction rates of 37.1 and 35.1%, respectively. These findings show that food addiction, self-esteem, and emotional eating are key predictors of disordered eating attitudes in the ANN model.

**Table 6:** Artificial neural networks multilayer perceptron layer levels.

Predictor		Predicted			
		Hidden layer 1		Output layer	
		H(1:1)	H(1:2)	EAT-26 = Under mean	EAT-26 = Over mean
Input layer	(Bias)	0.544	0.429		
	Gender = Female	0.371	−0.527		
	Gender = Male	0.469	−0.235		
	RSB Total	−0.538	0.536		
	EMAQ Negative	0.302	−0.371		
	YFAS Total	0.950	0.340		
Hidden layer 1	(Bias)			0.603	−0.409
	H(1:1)			−0.817	0.617
	H(1:2)			−0.579	0.044

RSB: Rosenberg Self Esteem Scale, EMAQ: The Emotional Appetite Questionnaire, YFAS: The Yale Food Addiction Scale.

**Figure 3:**
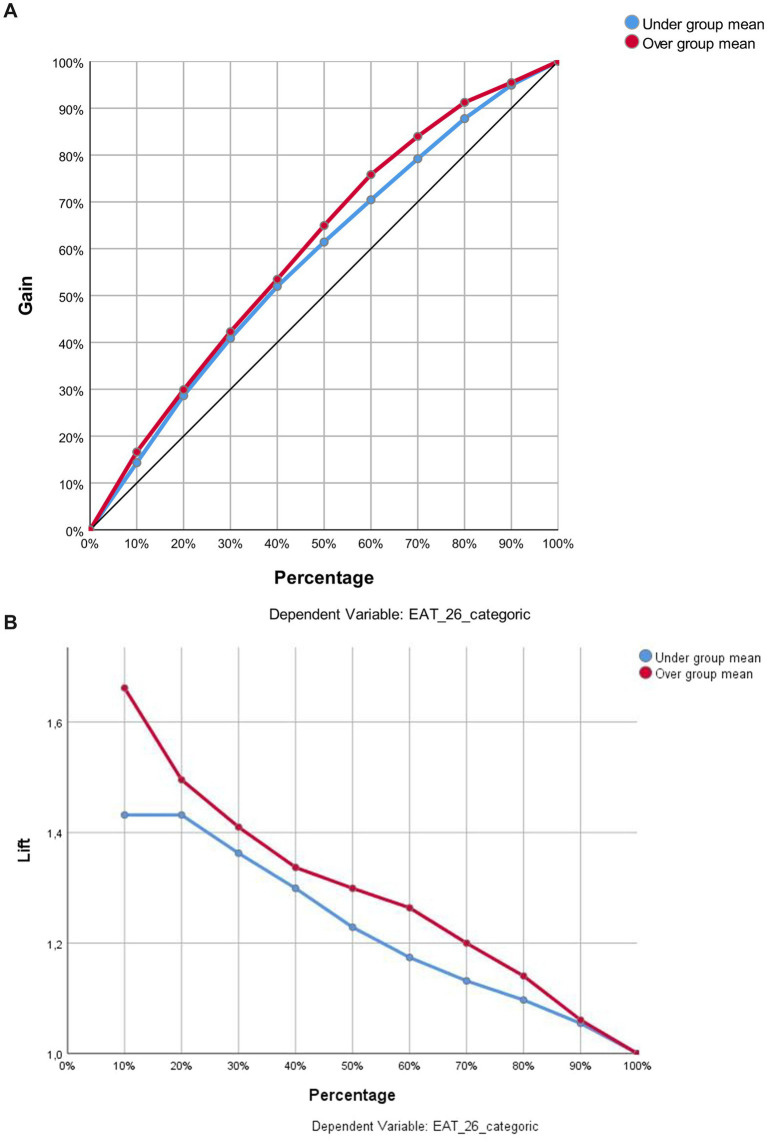
**(A,B)** Gain and lift charts for the classification performance of the model on eat-26 categories.

The Gain and Lift curves presented in Figure 3 show that the multi-layer perceptron artificial neural network model performed better than random classification. Consistent with the model’s prediction threshold, the sensitivity obtained from the H(1:1) neuron in the hidden layer (66.9%) is higher than that of the H(1:2) neuron (64.9%). These findings support the internal consistency of the model and show that the neurons in the hidden layer contribute significantly to the classification process. The lift values obtained being above 1 reveal that the model is more effective than the random model, especially in identifying the positive class (those with EAT-26 scores above the average). These results support the sufficient predictive capacity of the artificial neural network model and the possibility of classification based on psychological variables associated with EAT-26 (Figure 2).

## 4 Discussion

Food addiction and disordered eating behaviors represent significant psychiatric concerns, influencing both the clinical manifestation and the effectiveness of treatment. Eating disorders have gained global attention due to their connection with social, cultural, and psychological factors that influence eating attitudes and behaviors. These mental health issues, characterized by maladaptive eating behaviors and body image concerns, have risk factors such as early dieting, excessive weight loss habits, depression, body dissatisfaction, and social media influences (Tisko and Casas, 2023; Lonergan et al., 2020; Toklu Baloglu and Caferoglu Akin, 2024).

This study examined the effect of self-esteem, emotional appetite, and food addiction on eating attitudes in young adults aged 20–30. A total of 766 volunteers, including 171 females and 595 males, participated in this descriptive cross-sectional study. The BMI range was 13.0% underweight, 71.8% healthy weight and 15.2% overweight/obese. Participants of 97.0% of them stated that they eat outside the home, and 57.4% of them eat at school or workplace restaurants, the majority skip breakfast or lunch, 71.5% eat out of home 1–6 times a week, and 58.1% eat at school-work restaurants (Table 1). As the frequency of eating out increases, especially when fast food and high-calorie foods are preferred, gaining weight and the risk of obesity may increase. Each additional meal eaten at fast food or sit-down restaurant per week is associated with a measurable increase in BMI (Bhutani et al., 2018). According to Petrovics et al. (2021), obesity and overweight were observed more frequently in men, whereas women scored higher on eating patterns such as uncontrolled eating, cognitive restraint, and emotional eating, which were linked to reduced self-esteem. Gender appears to be the main determinant of uncontrolled and emotional eating, whereas BMI shows a comparatively weaker effect. Similar studies have demonstrated a positive association between food addiction, eating disorders, and BMI, with findings indicating that women face a greater risk than men regarding both food addiction and eating disorders (Şengör and Gezer, 2019).

The relationship between eating behavior, emotional appetite, and food addiction is complex and multifaceted. Emotional appetite refers to eating in response to emotional cues rather than hunger. It is emphasized that individuals with a high tendency toward emotional eating are more likely to exhibit symptoms of food addiction (Borisenkov et al., 2020; Sanlier et al., 2017), and that emotional eating is positively associated with food addiction (Borisenkov et al., 2020). People experiencing stress and depression often resort to emotional eating, which can increase their risk of developing food addiction (Fernández et al., 2022). The positive and significant relationship among eating-related variables (eating styles, binge eating and bulimia) with food addiction was demonstrated (Escrivá-Martínez et al., 2023). Low self-esteem is linked to less healthy eating attitudes and a greater risk of food addiction (Sanlier et al., 2017).

In this study, the EAT-26 eating concern subscale was found to be significantly associated with educational level, BMI, smoking, skipping snacks, and eating out. Furthermore, the EAT-26 dieting subscale was significantly related to BMI, while the social pressure subscale was negatively associated with educational level and BMI. The total EAT-26 score was also significantly associated with gender and BMI (*p* < 0.01; *p* < 0.05) (Table 3). Our results are similar to those of other studies (Kim et al., 2016; Magni et al., 2024). Emotional eating acts as a precursor to food addiction and is often an maladaptive coping strategy for emotional distress (Bozkurt et al., 2024; Fernández et al., 2022; Rossi, 2025). Uncontrolled eating and frequent thoughts about food can make young people more vulnerable to addictive eating habits by mediating the relationship between emotional states and food addiction (Fernández et al., 2022; Rossi, 2025). Psychological distress (stress, depression, anxiety) can increase the likelihood of food addiction by encouraging emotional and uncontrolled eating behaviors (Brytek-Matera et al., 2021; Skinner et al., 2021). Mindful eating practices are associated with lower rates of food addiction, suggesting that interventions targeting awareness may help reduce risk (Kaya Cebioğlu et al., 2022). This situation highlights the critical role of cognitive factors in directing maladaptive coping mechanisms such as emotional eating. Furthermore, emotional eating can often serve as a precursor to excessive eating behaviors stemming from food addiction. In particular, emotional eating triggered by negative emotional states is considered a significant predictor of food addiction among adolescents (Rose et al., 2018).

The eating concern subscale was negatively correlated with RSB, EMAQ negative and YFAS, while the restriction subscale was positively correlated with EMAQ positive, the social pressure subscale of EMAQ negative and EMAQ positive, the EAT-26 score was significantly associated with RSB, EMAQ negative, and YFAS (Table 4). A study has shown that EAT-26 is positively correlated with EMAQ negative scores, indicating that individuals at higher risk of eating disorders are more likely to engage in negative emotional eating behaviors (Barnhart et al., 2024). Emotional eating triggered by negative emotions seems to play a significant role in both the onset and persistence of disordered eating behaviors. Our findings indicate that emotional appetite influences both eating behaviors and food addiction. Previous research has also shown that eating behavior, shaped by physical, psychological, and social factors, impacts individuals’ dietary habits and overall health (Dugué et al., 2016; Alexatou et al., 2025). Adolescents with low self-esteem may use food as a coping mechanism, leading to disordered eating patterns (Sanlier et al., 2017). Studies have found that EAT-26 scores are particularly positively associated with food addiction and emotional eating in response to negative emotions (Cinelli et al., 2020; Sanlier et al., 2017; Sanlier et al., 2017). The YFAS has been reported to be positively correlated with EMAQ-negative scores, indicating that individuals with higher levels of food addiction are more likely to exhibit emotional eating behaviors in response to negative affect. This suggests that emotional eating plays a role in both the development and maintenance of food addiction (Burrows et al., 2018; Sanlier et al., 2017; Barnhart et al., 2024).

The findings of this study reveal that psychosocial variables such as self-esteem, emotional appetite, and food addiction are significantly related to eating behavior. In particular, it shows that an increase in emotional appetite is significantly related to food addiction and problematic eating behavior, while a decrease in self-esteem is significantly related to unhealthy eating behavior. Eating attitudes emerge as a decisive factor in explaining eating behaviors in young adults, based on their meaningful relationships with these psychosocial variables. Mental images play a role in the development of various psychological disorders and show a strong relationship with emotional processes. In the context of eating disorders, negative emotions and the mechanisms used to regulate these emotions are among the key determinants (Dugué et al., 2016; Alexatou et al., 2025). Although evidence highlights a strong relationship between emotional appetite, eating behaviors, and food addiction, this does not mean that all individuals who exhibit emotional eating tendencies will develop food addiction. Factors such as resilience, coping strategies, and social support can reduce these risks.

A multivariate generalized linear model showed that gender EMAQ negative, and YFAS total had statistically significant effects on EAT-26 (Table 5). Emotional states such as stress, loneliness, and boredom are strongly associated with overeating, especially among adolescent girls. Women generally report higher rates of eating addiction, emotional eating, and disordered eating behaviors (Petrovics et al., 2021; Skinner et al., 2021). Body image and subjective body shape also influence self-esteem and eating attitudes, particularly in girls (Petrovics et al., 2021). In this study, the effect of gender on EAT-26 scores was found to be statistically significant. Women’s EAT-26 scores were significantly lower than men’s, suggesting that unhealthy eating attitudes may be more pronounced among men and that this group may represent a potential risk in terms of eating attitudes. This result may be attributed not only to individual differences but also to broader social, cultural, and environmental factors. The predominance of male participants in the sample should be considered when interpreting gender-related findings. However, the inclusion of gender in the GLM model helped to partially control for this imbalance, contributing to a more reliable interpretation of the results. Adolescents’ eating behaviors are influenced by various factors such as peer pressure, media exposure, and family dynamics, and these factors can either reduce or intensify the impact of self-esteem and emotional states on eating habits.

Disordered eating attitudes (as measured by EAT-26) are most strongly linked to higher BMI, female gender, lower self-esteem, negative emotional eating, and food addiction. Social and educational factors also play roles, especially in how individuals respond to social pressure and snacking cues. Interventions should consider these interconnected psychosocial and demographic factors for effective prevention and treatment. Disordered eating attitudes (as measured by EAT-26) are most strongly linked to higher BMI, female gender, lower selfesteem, negative emotional eating, and food addiction. The risk of disordered eating is best predicted by the interaction of BMI, gender, and age, rather than any single factor alone (Jahrami et al., 2019). Food addiction and negative emotional eating are strong, independent predictors of disordered eating attitudes, often co-occurring and more prevalent in women and those with higher BMI (Bozkurt et al., 2024; Rose et al., 2018; Yua et al., 2018; Pape et al., 2021). Higher self-esteem can protect against the negative impact of high BMI and body-related shame on disordered eating (Petrovics et al., 2021; Razmus et al., 2023). Disordered eating attitudes (as measured by EAT-26) are most strongly linked to higher BMI, female gender, lower self-esteem, negative emotional eating, and food addiction. Social and educational factors also play roles, especially in how individuals respond to social pressure and snacking cues. Interventions should consider these interconnected psychosocial and demographic factors for effective prevention and treatment.

Considering that junk food products, ultra-processed food, fast food etc. are products with a low nutritional value and are hypercaloric, with the risk of causing a food addiction to develop and significantly alter the state of health. Especially when these types of food predominate in the diet and are accompanied by an unhealthy lifestyle (Mititelu et al., 2023). The higher energy density and oro-sensory characteristics of ultra-processed foods, fast food, etc., may also allow for greater energy intake in a shorter period of time. However, increased unhealthy feeding rates may lead to an increased energy intake, potentially due to a delayed signaling of satiety (Teo et al., 2022).

## 5 Conclusion and recommendation

As a result, statistically significant correlations were found between EAT-26, Rosenberg RSB, YFAS, and EMAQ. The findings indicate that low self-esteem is associated with problematic eating attitudes and food addiction, while increased levels of negative emotional eating are significantly associated with both unhealthy eating attitudes and food addiction. According to GLM and artificial neural network analyses, the variables with the strongest influence on eating attitudes are food addiction and negative emotional appetite. Model outputs revealed that these psychological variables significantly affected EAT-26 scores and that eating attitudes can be evaluated as a multidimensional psychosocial structure. The findings suggest that eating attitudes may be an important determinant in understanding the risk of food addiction among young adults. In this context, low self-esteem, negative emotional eating tendencies, and high levels of food addiction are important factors associated with unhealthy eating behaviors. A better understanding of the relationships between these variables can contribute to the promotion of healthy eating habits and the development of intervention programs. In particular, it can be said that focusing on emotional appetite and food addiction is important in education and intervention programs aimed at preventing eating disorders. Low self-esteem and emotional eating are significant risk factors for food addiction and disordered eating behaviors. These relationships are complex and often reinforce each other, highlighting the importance of addressing psychological and emotional factors in interventions.

Food addiction, disordered eating attitudes, low self-esteem, and emotional appetite are deeply interconnected. Addressing emotional and cognitive factors especially self-esteem and emotional eating can be crucial for preventing and treating food addiction and related eating disorders.

## 6 Limitations and strengths

A notable strength of this study is its status as one of the limited investigations exploring the links between eating behavior, emotional appetite, and food addiction within a traditionally structured country like Turkey. The research is based on a large male sample and employs multidimensional analyses. The study provides detailed insights into the associations between variables such as age, gender, education, tobacco and alcohol use, body mass index, self-esteem, emotional eating, and food addiction, and eating attitudes. Notably, eating attitudes were identified as an important risk factor among male individuals. The overlap between the results of two different modeling approaches, GLM and ANN, enhances the reliability of the findings. The Artificial Neural Network (ANN) model, in particular, captured the links between eating attitudes and self-esteem, emotional appetite, and food addiction not only in a linear context but also in a more powerful and flexible way through its multi-layered, learning-based structure. The model outputs revealed the effects of these variables on the EAT-26 with high classification accuracy.

Nevertheless, the cross-sectional nature of the study restricts the ability to draw causal inferences. In addition, reliance on self-reported height and weight data may have introduced potential measurement errors. We acknowledge that BMI values were self-reported, which may introduce some measurement bias. However, self-reported BMI is widely used in large-scale epidemiological and nutrition studies and has been shown to be highly correlated with objectively measured BMI. Given the large sample size and the nature of the data collection process, direct anthropometric measurements were not feasible. Therefore, we decided to retain BMI in the analyses while clearly acknowledging this limitation in the manuscript. The predominance of male participants in the sample may also reduce generalizability. The study was conducted on a sample of individuals aged 19–30 living in a large city, which restricts the generalizability of the findings to all age groups in Turkey. Therefore, it is important to replicate the study in other regions/countries.

## Data Availability

The datasets presented in this study can be found in online repositories. The names of the repository/repositories and accession number(s) can be found in the article/supplementary material.
